# Digital Adherence Technologies and Mobile Money Incentives for Management of Tuberculosis Medication Among People Living With Tuberculosis: Mixed Methods Formative Study

**DOI:** 10.2196/45301

**Published:** 2023-04-12

**Authors:** Angella Musiimenta, Wilson Tumuhimbise, Esther Atukunda, Aaron Mugaba, Sebastian Linnemayr, Jessica Haberer

**Affiliations:** 1 Faculty of Computing and Informatics Mbarara University of Science and Technology Mbarara Uganda; 2 Angels Compassion Research and Development Initiative Mbarara Uganda; 3 Faculty of Medicine Mbarara University of Science and Technology Mbarara Uganda; 4 Department of Economics, Sociology, and Statistics Rand Corporation Santa Monica, CA United States; 5 Medicine Department Harvard Medical School Boston, MA United States; 6 Center for Global Health Massachusetts General Hospital Boston, MA United States

**Keywords:** digital adherence technologies, real-time monitoring, SMS reminders, mobile money, financial incentives, tuberculosis, medication adherence, user-centered approach, mobile wallet, support medication, mobile phone

## Abstract

**Background:**

Although there is an increasing use of digital adherence technologies (DATs), such as real-time monitors and SMS reminders in tuberculosis medication adherence, suboptimal patient engagement with various DATs has been reported. Additionally, financial constraints can limit DAT’s utility. The perceived usefulness and the design mechanisms of DATs linked to mobile money financial incentives for tuberculosis medication management remain unclear.

**Objective:**

The aim of this study is to describe the perceived usefulness and design mechanisms for a DAT intervention called My Mobile Wallet, which is composed of real-time adherence monitors, SMS reminders, and mobile money incentives to support tuberculosis medication adherence in a low-income setting.

**Methods:**

This study used mixed methods approaches among persons with tuberculosis recruited from the Tuberculosis Clinic in the Mbarara Regional Referral Hospital. We purposively sampled 21 persons with tuberculosis aged 18 years and older, who owned cell phones and were able to use SMS text messaging interventions. We also enrolled 9 participants who used DATs in our previous study. We used focus group discussions with the 30 participants to solicit perceptions about the initial version of the My Mobile Wallet intervention, and then iteratively refined subsequent versions of the intervention following a user-centered design approach until the beta version of the intervention that suited their needs was developed. Surveys eliciting information about participants’ cell phone use and perceptions of the intervention were also administered. Content analysis was used to inductively analyze qualitative data to derive categories describing the perceived usefulness of the intervention, concerns, and design mechanisms. Stata (version 13; StataCorp) was used to analyze survey data.

**Results:**

Participants expressed the perceived usefulness of the My Mobile Wallet intervention in terms of being reminded to take medication, supported with transport to the clinic, and money to meet other tuberculosis medication–related costs, all of which were perceived to imply care, which could create a sense of connectedness to health care workers. This could consequently cause participants to develop a self-perceived need to prove their commitment to adherence to health care workers who care for them, thereby motivating medication adherence. For fear of unintended tuberculosis status disclosure, 20 (67%) participants suggested using SMS language that is confidential—not easily related to tuberculosis. To reduce the possibilities of using the money for other competing demands, 25 (83%) participants preferred sending the money 1-2 days before the appointment to limit the time lag between receiving the money and visiting the clinic.

**Conclusions:**

DATs complemented with mobile money financial incentives could potentially provide acceptable approaches to remind, support, and motivate patients to adhere to taking their tuberculosis medication.

**Trial Registration:**

ClinicalTrials.gov NCT05656287; https://clinicaltrials.gov/ct2/show/NCT05656287

## Introduction

Tuberculosis is a major public health concern that kills more people annually than HIV and malaria combined [1]. Suboptimal tuberculosis medication adherence can lead to treatment failure, development of drug-resistant tuberculosis, and secondary transmission [2-4]. Importantly, treatment for multidrug-resistant (MDR) tuberculosis is difficult to tolerate, prolonged, and expensive. Although there is an increasing use of digital adherence technologies (DATs), such as real-time monitors and SMS reminders, in tuberculosis medication adherence, research to date reports mixed results [5]. Differences in findings may relate to the extent of patient involvement in technology development, context, culture, and technology exposure. The World Health Organization (WHO) calls for assessment and mapping of potential users of DATs to support adoption and user engagement [6]. Additionally, despite their potential, data supporting the impact of DATs on treatment success and mortality are limited [7,8], and suboptimal patient engagement with various DATs for tuberculosis has been reported [9,10].

Our recently completed pilot randomized controlled trial demonstrated that real-time adherence monitors and SMS reminders are feasible and acceptable among persons with tuberculosis in southwestern Uganda; in particular, participants found the technologies are helpful in reminding and motivating medication adherence as well as being an important source of social support [11]. We also found a potential role for real-time monitoring linked to SMS reminders to improve tuberculosis treatment adherence; however, financial constraints were a notable limitation [12], suggesting a potential role for financial incentives.

The WHO End tuberculosis strategy recommends using reimbursements and social protection schemes to lower the social and economic burden of tuberculosis [13] and address social determinants of health. Although research in this area is limited, a monthly financial incentive package improved tuberculosis treatment success (presumably completion of 6 months of treatment) and reduced loss to follow-up among low-income people in Nigeria [14]. A recent systematic and meta-analysis literature review composed of only 8 studies indicates that money transfer interventions for patients initiating tuberculosis treatment may improve clinical outcomes [15]; for instance, in Brazil, money transfers improved rates of tuberculosis cure by 82% [16]. The review called for more research specifically in low-income countries where financial incentives may have the strongest effect. Importantly, the impact of these studies may be limited by providing incentives face-to-face; they are thus restricted by geographical boundaries, are time-consuming, and involve transport costs.

Currently, there are over 5.48 billion unique mobile phone users globally and nearly 5.04 billion mobile internet users [17]. In Uganda specifically, mobile phone reception is available across the vast majority of the country, including many rural areas and among economically disadvantaged populations [18]. The rapid evolution of mobile phones has enabled a mobile payment platform (often known as mobile money), which enables microbanking financial transactions (eg, sending, saving, paying, and receiving money) possible using simple mobile phones that are independent of smartphone capabilities or internet access. Indeed, use of “mobile money” (money accessed through mobile phones) services is nearly ubiquitous in Uganda and in many transitional countries [19]. Many people in Uganda are increasingly relying on mobile money as they lack access to formal banking services—more than 23.5 million people have mobile money subscriptions [19]. Among the underserved populations, mobile money has enabled routine payment of health insurance and improved access to family planning [20], as well as enabled saving and making payment for pregnancy-related care [21]. Mobile services could also be used to provide financial support for tuberculosis treatment adherence; however, research in this area is lacking.

Optimal design of mechanisms to incorporate financial support into DATs for tuberculosis treatment would include input from people with tuberculosis. User-centered design (UCD; sometimes known as human-centered design) involves systematically eliciting input from prospective users to guide the design of interventions [22] rather than assuming that designers know what users need. Incorporating insights from prospective users may result in context-specific and culturally acceptable interventions that can most effectively meet the needs of the end users [23]. This paper describes the perceived usefulness, concerns, and designed opportunities regarding a DAT intervention called *My Mobile Wallet* that is composed of real-time adherence monitors, SMS reminders, and mobile money incentives to improve tuberculosis medication adherence in a low-income setting.

## Methods

### Study Design and Setting

This study used a convergent mixed methods study design because we concurrently collected qualitative and quantitative studies, analyzed qualitatively and quantitative data independently, and interpreted the results together [24]. Our study participants were persons living with drug-sensitive tuberculosis (persons with tuberculosis) recruited from the Tuberculosis Clinic in the Mbarara Regional Referral Hospital (MRRH) in southwestern Uganda, which provides care to an estimated 600 persons with tuberculosis annually. At the Tuberculosis Clinic, the newly diagnosed persons with tuberculosis receive free tuberculosis medication and are counseled about the benefits of tuberculosis medication. Directly observed therapy is not used for monitoring medication adherence at MRRH due to the costs involved for both persons with tuberculosis and the health care workers. Instead, persons with tuberculosis are given the 2HRZE/4HR tuberculosis treatment regimen (2HRZE: isoniazid, rifampin, pyrazinamide, and ethambutol for 2 months; 4HR: isoniazid plus rifampin for 4 months) every 2 weeks for the first 2 months, at which time they return to the Tuberculosis Clinic for a sputum conversion check. Those who become smear negative continue with isoniazid and rifampin only for a period of 4 additional months in the continuation phase. Those with positive test result receive GeneXpert to exclude rifampicin resistance; subsequent treatment is then individualized. Treatment is also extended up to a full year to cater for missed medication pickups or doses.

### Selection of Study Participants

Between May 2022 and June 2022, we purposively recruited 21 participants (persons with tuberculosis) according to the following inclusion criteria: (1) being aged 18 years and older, (2) being infected with tuberculosis and undergoing a first-line 6-month course of standard antituberculosis therapy (per above) and counseling per clinic records, (3) owning a cellphone for personal use, and (4) being able to use SMS text messaging. Participants who were unwilling or unable to give consent did not have a cell phone and were unable to use SMS text messages were excluded. A subset of participants for focus group discussions (FGDs) to achieve relatively balanced gender representation were purposively sampled. We also purposively enrolled 9 participants who used DATs in our previous, above-noted study [11] who had completed a first-line 6-month course of antituberculosis therapy. The selection of the 9 participants was purposive based on (1) our perceptions about their openness in discussing qualitative issues based on our previous experience with them in the DATs study, (2) the functionality of their phones at a time of calling them to request their study participation, (3) their proximity to MRRH where we recruited participants for this study, (4) as well as their willingness to participate in this study. The main aim of including the 9 participants in this study was to learn from their practical experiences unlike the other newly recruited 21 participants who had no practical experience in using DATs.

### Intervention Technology

**Figure 1:**
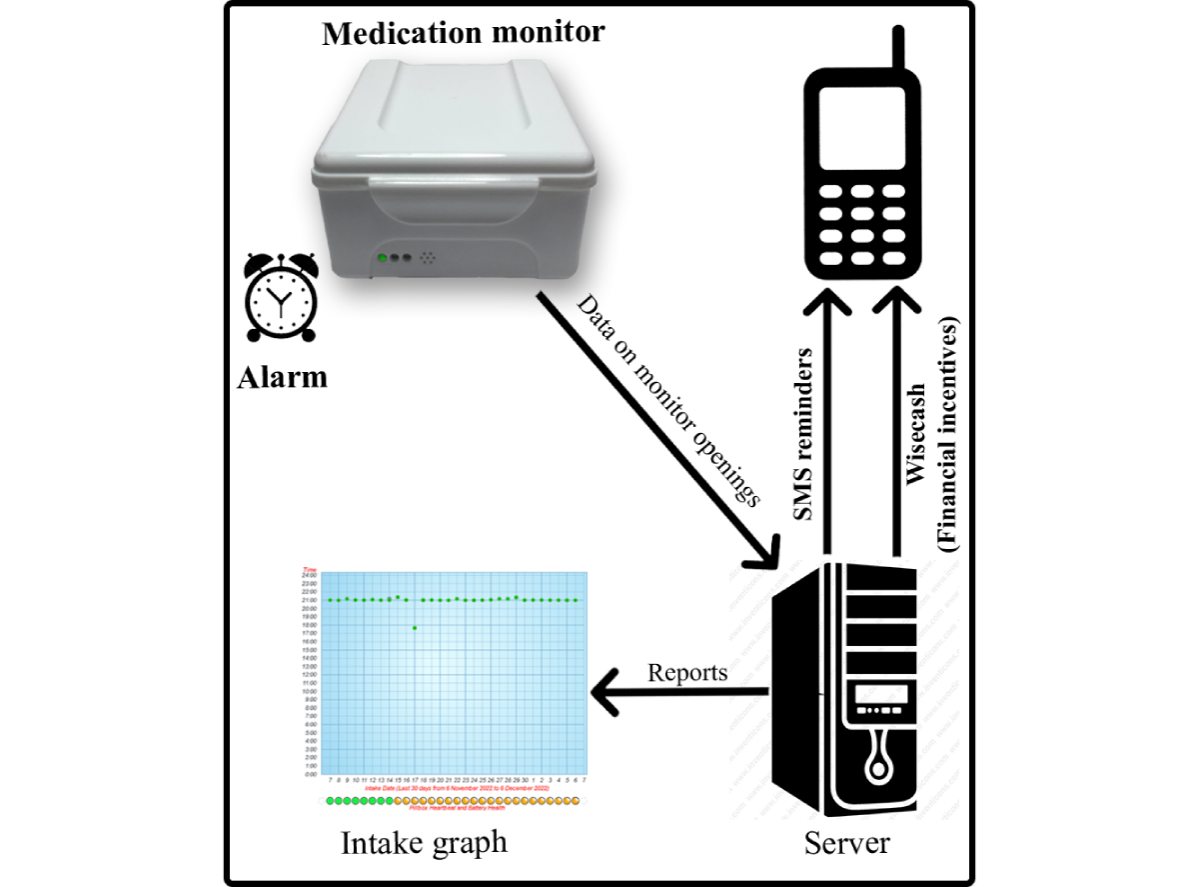
The My Mobile Wallet intervention diagram.

As indicated in Figure 1, the *My Mobile Wallet* intervention is composed of (1) a real-time medication monitor (Wisepill Technologies, Cape Town, South Africa) to monitor medication adherence, (2) SMS reminders to support persons with tuberculosis in taking their medication as prescribed, and (3) a *WiseCash* component for sending mobile money incentives for transport to the clinic and motivating medication adherence.

**Figure 2:**
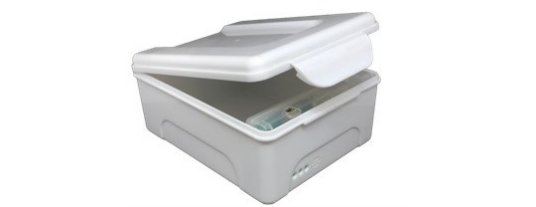
The evriMED dispenser container to easily hold a month’s supply of tuberculosis medication.

**Figure 3:**
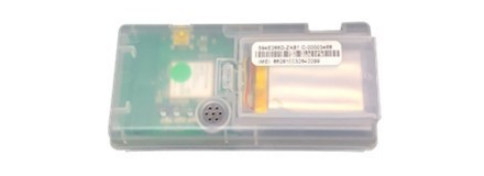
The evriMED electronic module slots into the container so that the indicator light-emitting diodes are visible through the front of the container.

The real-time adherence monitor (evriMED1000 internet-enabled pillbox) is a device that sends signals when opened (records a date-and-time stamp) as a proxy for taking medication. The evriMED1000 is a tabletop dispenser, designed to easily hold a month’s supply of tuberculosis medication. It is an affordable scalable tool, which allows real-time adherence management by automatically synchronizing with the Wisepill cloud service. The evriMED1000 dispenser consists of 2 hardware components, namely the medication container (Figure 2) and the electronic module (Figure 3). This modular design allows the electronic module to be reused and the container to be replaced if needed. The electronic module slots into the container so that the indicator light-emitting diodes are visible through the front of the container. An internal modem and SIM card enable the device to send a real-time mobile signal to a secure web server (hosted in South Africa). General packet radio service maintains the data in transit until the web server acknowledges receipt, which minimizes possible data loss because of power failure or lack of internet connectivity. In the event of inadequate mobile network coverage, the monitor stores openings in flash memory and sends them when the network becomes available. The monitor also transmits a daily “heartbeat” that indicates current battery life, remaining airtime balance, and signal strength as indication of its functionality. The monitor can be charged using electricity or a solar device. Its battery life is 6 months.

**Figure 4:**
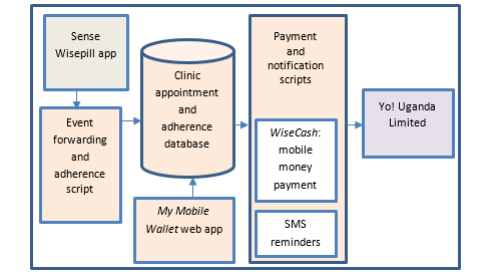
My Mobile Wallet functional diagram.

As indicated in Figure 4, *WiseCash* consists of five main elements: (1) “Sense Wisepill” which is a web-based software app that allows the configuration and management of the real-time adherence monitor, including linking participants to their respective real-time monitors; (2) an event forwarding and adherence software program that enables the receipt of the real-time monitor openings as adherence events from the Wisepill server; (3) a clinic appointment and adherence database to store and track participant data and adherence; (4) *My Mobile Wallet* web app to allow the management of participant clinic appointments and payments; and (5) payment and notification apps to implement the actual payments and send notifications to participants in the local language (Runyankole).

*WiseCash* includes a script that daily checks participant appointments, evaluates user adherence, and queues and makes payments and payment notifications to participants when due. Yo! Uganda Limited (Kampala, Uganda) is a technology solutions company that offers a single application program interface (API) to provide payments to SIM cards for 2 telecom providers (ie, MTN and Airtel). *WiseCash* is hosted by Xneelo Limited, a web-hosting company in South Africa. Additionally, Yo! Uganda Limited also provides a second API for the SMS reminders. A number of security elements are included in *WiseCash* including Yo! Payment platform passwords for the research team members to manage the *My Mobile Wallet* intervention; API keys with Wisepill data stored on a secure server; and a digital authorization by the research staff for payments triggered by the *My Mobile Wallet* intervention.

### Study Procedures

We first oriented each participant about the *My Mobile Wallet* intervention components by presenting to them an initial version of the intervention as described earlier. We explained and demonstrated how the real-time adherence monitor (Wisepill) works, including how it monitors medication and sends a signal to researchers every time it is opened, how the monitor makes an alarm to remind medication taking, and how to open and close it to put in or retrieve medication. Participants who had never used the monitor were asked to practically demonstrate how it works. We then demonstrated how the intervention could send daily SMS reminders to remind persons with tuberculosis take medication. We also explained how the intervention will send monthly mobile money (US $8) to phones belonging to persons with tuberculosis for transporting them to the clinic at recruitment until the end of their 6-month treatment and monthly medication adherence incentives (US $1.5) to those who meet a ≥90% medication adherence as ascertained from a real-time adherence monitor. The ≥90% medication adherence was considered because adherence less than 90% has been reported as a risk factor for unfavorable treatment outcomes [25].

### Data Collection and User-Centered Technology Development

We used FGDs to solicit participants’ (persons with tuberculosis and patients who had tuberculosis) perceptions about the initial version of *My Mobile Wallet* intervention. The development of questions for the FGDs (Multimedia Appendix 1) was informed by the Unified Theory of Acceptance and Use of Technology model with specific focus on participants’ perceived usefulness of the intervention, as well as the anticipated challenges in using the intervention [26]. Subsequent versions of the intervention were developed using a UCD approach, which is an iterative technology design process in which users’ needs are continuously solicited and included in the design and development process in order to generate products that can easily work in specific contexts [27,28].

With UCD, technologies are designed and tailored toward users’ needs (rather than the users having to adjust their behaviors to accommodate the technology) to enhance usefulness, usability, and use of technology. An iterative refined version of the intervention was developed based on feedback from participants until the beta version of the intervention suited their needs. Specifically, we conducted a series of 5 FGDs over a period of 6 months to develop the intervention. FGDs were focused on assessing how participants would feel about using the intervention, including (1) anticipated benefits and barriers; (2) usage of mobile phones, mobile money, and SMS text message; (3) preferred content of mobile phone messages; and (4) appropriateness of the intervention for all persons with tuberculosis in the region versus those with demonstrated poverty (eg, means testing). FGDs lasted between 90 and 120 minutes and were conducted in Runyankole, which is digitally recorded, transcribed, and translated to English. Participants tested the initial version of intervention (eg, to withdraw money from it, receive SMS text message, and open the real-time monitor) and provided feedback. We also administered surveys (Multimedia Appendix 2) composed of both closed and open-ended questions. Information elicited by surveys include participants’ cell phone use, preferred frequent and content of SMS text message, timing for sending transport incentives, and anticipated barriers in using the intervention.

### Data Analysis

We used inductive content analysis [29] to derive categories describing and summarizing how participants perceived the intervention. Initially, authors A Musiimenta and WT reviewed and discussed 20% of transcripts for content relevant to participants’ perceptions about the intervention, anticipated benefits, and challenges. A Musiimenta and WT then assembled a codebook from the identified concepts, using an iterative process, which included developing codes to represent content, writing operational definitions, and selecting illustrative quotes. JH and SL reviewed and discussed the codebook. Following completion of the codebook, A Musiimenta and WT applied codes using NVivo (version 11; QSR International). Differences in coding were harmonized through discussion. We followed the Consolidated Criteria for Reporting Qualitative Research (COREQ-2) [30] in reporting qualitative results. A Mugaba and A Musiimenta used Stata (version 13; StataCorp) to describe study participants, cell phone use, and preferences regarding the intervention.

### Ethics Approval

The institutional review committees of Mbarara University of Science and Technology (MUST-2021-102) and the Uganda National Council for Science and Technology (HS1688ES) approved this study. All participants provided signed informed consent before study participation.

## Results

### Participants’ Demographic Characteristics at Enrollment

Of 35 screened participants, 3 (9%) were excluded for not having a cell phone and 2 (6%) were excluded for being unable to use SMS text messages. Between May 2022 and June 2022, a total of 30 participants were enrolled in the study and participated in 5 FGDs (each FGD composed of 6 participants, totaling to 30) of whom 21 (70%) were still on tuberculosis treatment with median months on medication of 1 (IQR 1.0-3.5), while 9 (30%) were former persons with tuberculosis from our previous study with a median months after completing treatment of 30 (IQR 28.0-34.5) (Table 1). The majority of participants were living with HIV coinfection (n=26, 87%) and female (n=20, 67%). Most were in their early 40s (median age 44.5, IQR 32.3-52.5 years) and had not studied beyond primary level (n=19, 63%).

**Table 1:** Demographic characteristics.

Characteristics			Values (N=30)
Age (years), median (IQR)			44.5 (32.3-52.5)
Female, n (%)			20 (67)
Male, n (%)			10 (33)
**Education, n (%)**			
	None	3 (10)	
	Primary (P1 to P7)^a^	19 (63)	
	Ordinary level (Senior 1 to 4)	8 (27)	
Months on medication (N=21), median (IQR)			1 (1.0-3.50)
Months after tuberculosis treatment (N=9), median (IQR)			30 (28.00-34.50)
Positive HIV status, n (%)			26 (87)
No regular income (fixed wages or salary), n (%)			27 (90)

^a^In the Ugandan education system, primary (P1-P7) is often attended by 6- to 12-year-olds. Ordinary level is often attended by 13- to 16-year-olds.

### Survey Results

As indicated in Table 2, the majority of participants reported not sharing their phones (n=24, 80%), checking their phones for SMS text message more than once a week (n=24, 83%), preferring to receive daily SMS reminders to weekly SMS (n=23, 77%), and preferring to receive SMS reminders that are not easily related to tuberculosis (n=20, 67%). The majority of participants (n=25, 83%) preferred being sent mobile money transport incentives 1-2 days before clinic visit. Barriers anticipated in receiving money included automatic reduction of money by mobile phone service providers for participants who borrow money from the service providers (n=4, 13%), the possibility of using the money for other competing demands such as buying food (n=12, 40%), misusing the money by other people in case of phone sharing (n=4, 13%), and inability to read mobile money messages (n=8, 27%).

**Table 2:** Survey results.

Characteristics		Values (N=30), n (%)
**Who else uses your phone?**		
	Spouse	3 (10)
	Family member	2 (7)
	Friends	1 (3)
	No one	24 (80)
**Check for SMS** **text messages** **at least once a week**		
	Never	3 (10)
	Less than often	2 (7)
	More than often	25 (83)
**Delayed from checking SMS** **text messages** **last week**		
	Phone not charged	6 (20)
	Phone used by somebody else	1 (3)
	No adequate signal	1 (3)
	Used by someone else	1 (3)
	Never delayed	21 (70)
**Preferred frequency of receiving SMS reminders**		
	Daily	23 (77)
	Weekly	7 (23)
**Preferred content for SMS** **text messages**		
	Not easily related to tuberculosis (eg, hello today)	20 (67)
	Easily related to tuberculosis (eg, take your tuberculosis drugs)	10 (33)
**When to send the incentive for transport**		
	A day before clinic visitation	15 (50)
	Two days before clinic visitation	10 (33)
	More 2 days before clinic visitation	5 (17)
**Barriers anticipated in receiving mobile money^a^**		
	Automatic reduction of money due to phone loan	4 (13)
	Using the money for other things due to competing interests	12 (40)
	Money ending in wrong hands due to phone sharing	4 (13)
	Inability to read mobile money messages	8 (27)
**People to benefit most from the intervention**		
	All persons with tuberculosis	4 (13)
	Persons with tuberculosis with demonstrated poverty (eg, lack of regular income)	26 (87)

^a^Participants could provide multiple responses.

### Qualitative Results

#### Perceived Usefulness of the Components of the My Mobile Wallet Intervention

As indicated in Table 3, some participants (particularly those who had used the real-time device in the prior study) reported that looking at the device and knowing that their medication taking is being monitored motivated them take medication on time. Participants perceived the real-time monitor as a friendly *companion* that can offer welcoming medication monitoring compared to human beings whose mood may be dictated by circumstances. Additionally, some participants perceived SMS reminders as useful for reminding patients to take medication especially for those who are initiating treatment because they are not yet accustomed to the behavior, those who lack social support in their homes, and busy patients who can easily forget. Participants reported that incentives for transport to the clinic could address the challenges that constrain medication adherence such as missing pill refills due to lack of money for transport, which often requires walking long distances to the clinic or borrowing money to get transport. Participants indicated that adherence incentives could instill inward motivation to take medication well in order not to miss the money to buy basic necessities of life (such as food) and consequently improve medication adherence.

**Table 3:** Technology description and participants’ perceived usefulness of the technology.

Technology description	Perceived usefulness
Real-time adherence monitor (Wisepill device) is a “smart” pillbox that records a date-and-time stamp for each opening as a proxy for medication ingestion.	*This device helped me a lot when I was still taking my tuberculosis medication. From the time you gave it to me, I started taking my medication well because every time I would look at it, I would be automatically reminded about my obligation to take medication on time. And because you told me it reports the way I take medication, I made sure I was taking my medication on time to maintain a good report.*
The data are sent to a central server by general packet radio service (as is standard for most data transmission) with SMS backup in the case of poor cellular network availability. The device monitors medication adherence and also has an alarm that sounds at the exact time of taking medication to remind medication taking.	*Sometimes being monitored by the device may save the persons with tuberculosis from interfacing with health care workers or treatment supporters who are rude in talking and handling patients. Some people are naturally rude, while others become rude due to personal problems. Imagine if you have to swallow your medication before such a rude person? For instance, in a case where a patient missed some dose for unavoidable reasons, the patient may fear to be confronted by a rude person and instead decide not to turn up medication taking. But when you have this device, you are not worried of such.*
Daily SMS reminders sent to persons with tuberculosis 30 minutes before their time for taking medication to enable them to prepare to take medication.	*The SMS reminders I was receiving from the study played a big role of reminding me to take medication. When I had misunderstandings with my wife, she could no longer remind me take my medication or even provide food and drinks on time to enable me take my medication. Much as no one cared for me at home or gave me counseling, because of receiving SMS reminders, I was confident that someone out there was with me in the struggle and cared for me, so, I kept taking my medication on time, and I did not feel a lot of stress.*
*WiseCash* informs of transport incentives. Monthly mobile money for transport (US $8) to the clinic is sent to the patient’s phone a day before the clinic appointment.	*Assisting persons with tuberculosis with transport is important because, for example, personally, I always struggle to get transport for my clinic appointment because I have no source of income. I am serious with taking medications but I am challenged with lack of money for transport to come to the clinic for refill. Sometimes, I fail to honor my appointments because I have no one to help me and it is difficult to walk for long distances with this sickness because of chest pain. This makes me spend some days without taking medication.*
*WiseCash* informs of adherence incentives. A monthly medication adherence incentive of US $1.5 sent with the transport funds to persons with tuberculosis that meet a ≥90% medication adherence as ascertained from a real-time electronic adherence monitor.	*It is good as it doesn’t only help persons with tuberculosis but it also motivates those who have not tested to have themselves tested when they know about the incentives. I know some people who are sick out there but because they are poor, they have not come out for treatment because they think it is costly to come for testing, undergo treatment, and take the required foods and drinks. This money can be used to buy food for taking medication because this medication makes you weak and dizzy if you take it on an empty stomach. Actually, I would dodge it whenever I had no food because it made me eat a lot which means I was spending more on food.* *Giving incentives to good adherers is useful because it will make persons with tuberculosis compete and focus on taking medication in order to be incentivized and in the process, they will adhere to their medication and eventually have their health improved. I remember when we were studying, we used to work hard in order to get presents for performing well in class, so the same thing applies to this approach. Even persons with tuberculosis who have been poor adherers are likely to start taking well because they do not want to miss that money.*

#### Concerns About My Mobile Wallet Intervention and Design Considerations

Concerns and design opportunities reported by participants are described in Tables 4 and 5. They include (1) possibilities of unintended status disclosures triggered by seeing the SMS reminders or real-time monitor or hearing the alarm from the monitor; (2) using the money meant for transport to the clinic for other competing demands; and (3) technology fatigue mainly emanating from daily SMS text message and monitor alarms. Design considerations adopted in response to the concerns include using language in the SMS text message that is confidential and not easily related to tuberculosis, allowing participants to decide if they want to have the alarm on the monitor activated, sending transport refund a day before the appointment to limit the time lag between receiving the money and visiting the clinic, and sending the money to patient’s personal phone to avoid possibilities of money being misused by other people. Other modifications added during the iterative phase of technology development are outlined in Table 5.

**Table 4:** Participants’ concerns and design opportunities.

Concerns	Illustrations	Design considerations
Possibility of unintended status disclosure	*Someone might read the SMS reminder sent to your phone to remind you to take tuberculosis medication or might open the device and realize that it contains tuberculosis medication. They may in the process get to know your tuberculosis status and start spreading the rumor in the whole village, treating you badly, and avoiding you.* *The alarm on the Wisepill device might attract people’s attention and result in unintended status disclosure.*	Using SMS reminders and mobile money messages which are not easily linked with tuberculosis preferred to reminders which can easily be linked to tuberculosis. Participants decide whether they want the alarm on the Wisepill device on or off.
Using money meant for transport to the clinic, for other competing interest	*My worry is that this money will be used for different purposes, for example, a patient has received this money when he or she has some financial problems at home, they will just focus on addressing that problem using the money that is meant for transport to the clinic and when the time for hospital visitation comes they find themselves helpless.*	Sending the money a day before rather than several days before the appointment could reduce the possibility of using it for other purposes. Giving transport refund when persons with tuberculosis come to hospital than sending them money before (this option was not considered).
Money ending in wrong hands due to phone sharing	*If that money is sent during unfortunate times when the person I share the phone with is having some financial challenges or emergencies, the person will just use the money for themself and ignore me.* *Some husbands especially those who are alcoholics may not give the money to their wives if it is sent on their phones.*	Considering personal ownership at recruitment so that money is sent to the phones owned by persons with tuberculosis.
Technology fatigue from SMS reminders and device alarms	*Since both SMS reminders and device alarms are both meant to remind persons with tuberculosis take their medication, it might be too much for them to have both.*	Participants are given an opportunity to choose whether or not they want to receive medication-taking alarms from the device on top of receiving SMS reminders.

**Table 5:** Other intervention design issues considered.

Initial design	Refinement or addition
Mobile money messages in English language	A language option was added so that participants can be notified in the local language (Runyankole) to cater for participants who cannot read English.
No default payment date	To cater for instances where no appointment has been set, a default payment date was added as the 25th day of the month.
No arrangement for pending payments	The ability to cancel a payment was added. Cancelling a payment is effective if a payment is still pending (after 2 hours of failed attempts, eg, in case of invalid phone numbers) or if there is a problem with the transaction at the service provider.
No notifications for payment delivery status	To keep track of the mobile money transactions and notifications, an email notification to the *My Mobile Wallet* administrator for successful or unsuccessful payment was added.
Absence of a log file on the administrator’s side	A link to a log file was added to the payment screen in order to provide history of transactions and analyze the performance of the payment and notification functionality.

## Discussion

### Principal Results

The *My Mobile Wallet* intervention composed of real-time adherence monitor, SMS reminders, and mobile money incentives was perceived as useful in supporting tuberculosis medication adherence in a low-resource setting. Participants expressed the perceived usefulness of the My Mobile Wallet intervention in terms of being reminded to take medication, supported with transport to the clinic, and money to meet other tuberculosis medication–related costs, all of which were perceived to imply care, which could create a sense of connectedness to health care workers. This could consequently cause participants to develop a self-perceived need to prove their commitment to adherence to health care workers who care for them, thereby motivating medication adherence.

Through a UCD process, we learned that the main concerns were (1) unintended status disclosure triggered by seeing the real-time monitor or hearing the alarm from the monitor or seeing the SMS reminders and (2) using the money meant for transport to the clinic for other competing demands. The main design considerations that arose in the process were (1) using language in the SMS text message that is confidential and not easily related to tuberculosis and (2) sending transport refund a day before the appointment to limit the time lag between receiving the money and visiting the clinic.

### Limitations

The main limitation of this study is that we asked participants about perceptions before they could use the intervention in real life, although 9 participants (30%) had used the real-time monitor and SMS text message in our prior study. Although we practically demonstrated the use of the intervention to all participants, 21 (70%) participants were not able to describe actual experiences using the device or medication-related SMS reminders or financial incentives as part of their daily routine.

### Comparison With Prior Work

Participants who had used the real-time monitor felt being *cared for* as a result of being monitored and sent an SMS reminder. This created a sense of connectedness to health care workers and consequently caused participants to develop a self-perceived need to prove their commitment to adherence to health care workers who cared for them. Participants referred to the real-time monitor as a *friendly companion* that can potentially offer welcoming medication monitoring compared to human beings whose mood may be dictated by circumstances.

Given the prevailing stigma and discrimination that is associated with living with tuberculosis in Uganda [31], such positive perceptions could empower participants to cope with social isolation and other challenges of having tuberculosis, especially among those who do not have adequate social support, those initiating treatment who are not yet accustomed to taking medication, and those living with HIV coinfection who are more likely to experience double stigma from both tuberculosis and HIV. Experiences of unintended HIV status disclosure were reported by the people living with HIV or AIDS who used the real-time device and SMS reminders in the same setting [32]. Noteworthy, however, these technologies can also facilitate intended HIV status disclosure in order to get social support [33]. To reduce the potential effects of the tuberculosis status disclosure that can potentially emanate from using the intervention, strategies to ensure privacy and confidentiality (eg, using SMS text message that is not easily related to tuberculosis) and reduce stigma and discrimination were incorporated into the intervention.

Participants felt that providing financial incentives in the form of mobile money could relieve persons with tuberculosis of the transport burdens associated with clinic visitations for pill refill, thereby eliminating the need to walk long distances to the clinic or borrow money to get transport or miss pill refill appointments. Lack of transport to the clinic is one of the major challenges constraining tuberculosis medication adherence in Uganda [34]; 2 in every 10 people (21% of the population) live on US $1.25 a day or less [35], making saving for health difficult amid other immediate competing interests. To reduce the possibility of using the money meant for transport to the clinic for other competing demands, participants proposed sending transport refund a day before the appointment. Participants also reported that financial incentives could promote medication taking in order not to miss the money involved, which could be used to meet other costs related to tuberculosis treatment such as nutritional supplements and food. This is important given that in Uganda, tuberculosis or HIV high burdened country, over 53% of tuberculosis-affected households experience tuberculosis costs, which are above 20% of their annual household expenditures, and the main cost drives are nonmedical expenses such as transport to the clinic and food [36]. Because tuberculosis disproportionately affects the poorest in the population, its poverty-aggravating effects are felt more by those who are already vulnerable. Moreover, having tuberculosis is associated with other negative socioeconomic consequences, such as divorce, school interruptions for children, and social exclusion, in addition to the hardship of deteriorating health. Incentive-based interventions can potentially overcome the poverty-based structural barriers to tuberculosis treatment, although research in this area is limited. Incentive-based interventions can also potentially be integrated with routine health care especially given the evidence that an incentive as small as US $1 can improve tuberculosis cure and reduce loss to follow-up among persons with tuberculosis in rural Uganda [37]. This can potentially prevent drug resistance, thereby relieving the country of the financial burden associated with MDR tuberculosis treatment—Uganda has over 16,000 patients with MDR tuberculosis [35]. The country currently spends US $105 (on transport refund and food incentives) per month on each patient with MDR tuberculosis from the time they are discharged (after the initial 6 months of hospitalization) until treatment completion (1 year). This translates to a cost of US $1260 per patient for the 12 months of treatment. Importantly, treatment for MDR tuberculosis leads to productivity loss for patients and their caregivers, which negatively affects the economic development of the country.

### Conclusions

Using a mixed methods approach to collect both closed- and open-ended data, this study identifies important insights (about perceived usefulness, concerns, and design considerations) from patients with tuberculosis that can inform the development of real-time adherence monitoring technologies linked to mobile money–based financial incentives. In sum, real-time adherence technologies complemented with mobile money–delivered financial incentives could potentially provide acceptable approaches to remind, motivate, and support patients to adhere to taking their medication especially in settings where directly observed therapy short course is difficult to implement as well as low-resource settings where poverty-based structural barriers to tuberculosis treatment are prevalent.

As DATs proliferate, using user-centered approaches to understanding how they are perceived by users is critical to developing tailored interventions that are useful, usable, and acceptable in supporting tuberculosis medication adherence. Findings from this formative study enabled the design of the *My Mobile Wallet* to support tuberculosis care and treatment adherence, which is currently being implemented for initial feasibility and acceptability assessment. After this initial assessment, further refinements will be done before the technology can be further tested for large-scale feasibility, acceptability, and impact in a planned randomized control trial (NCT05656287).

## Appendix

Multimedia Appendix 1 Focus group discussion guide.

Multimedia Appendix 2 Social demographics survey.

## Abbreviations

API: application program interface
COREQ: Consolidated Criteria for Reporting Qualitative Research
DAT: digital adherence technology
FGD: focus group discussion
MDR: multidrug-resistant
MRRH: Mbarara Regional Referral Hospital
UCD: user-centered design
WHO: World Health Organization

## Data Availability

The data sets generated and analyzed during this study are available from the corresponding author on reasonable request.
