# Supplementary material for: Digital Adherence Technologies and Mobile Money Incentives for Management of Tuberculosis Medication Among People Living With Tuberculosis: Mixed Methods Formative Study
Source: JMIR Form Res. 2023 Apr 12;7:e45301. doi: 10.2196/45301 (PMC10134020; doi:10.2196/45301)
Supplement: Multimedia Appendix 1 [file formative_v7i1e45301_app1.pdf]

Title: *My Mobile Wallet*: An Intervention to Support Access to Tuberculosis Care and Medication Adherence in Rural Uganda

R21 Aim 1: Focus Group Discussion with TB patients for intervention development

Thank you for coming to this group discussion today. As we mentioned earlier, we are planning to carry out a study whose main goal is to improve TB medication adherence through *My Mobile Wallet*— a novel intervention that utilizes SMS texts to remind medication adherence, as well as monthly mobile money incentives for transport to TB clinic, and motivate medication adherence using a special pill container called Wisepill. The study will involve 242 TB patients receiving TB services from the TB clinic at Mbarara Regional Referral Hospital.

Here is a demo of the application of *My Mobile Wallet* which we hope to improve and give to TB patients *<practically show the current prototype (to withdraw money from it, receive SMS texts) then demonstrate the use of Wisepill device>*.We hope to enhance this prototype, and then learn if the monthly mobile money for transport and adherence incentives will motivate medication adherence among TB patients.

We are now going to ask you about the economic and behavioural challenges you experience in accessing TB treatment services and taking your medication on time, as well as your perceptions about the My Wallet Intervention.

There are no right or wrong answers, we just like to know what your thoughts are.

We will record your responses, so that we may write them down accurately at a later time. We will then delete the recording to protect your confidentiality.

Before we get started, do you have any questions for me?

What economic and behavioural challenges do you normally experience in accessing TB services and/or taking to your TB medication on time?

Probe: *transport to the clinic, long distances, poverty/lack of regular income, high transport costs, instrumental support, motivation, forgetfulness*

Perceptions of My Mobile Wallet (Mobile Money) Intervention

Read: *I would now like to ask you some questions about the* monthly mobile money incentives for transport to TB clinic and medication adherence.

How do you feel about an intervention that utilizes mobile money approach to provide transport to TB clinic, and motivate medication adherence?

RESEARCH ETHICS COMMITTEE
MBARARA UNIVERSITY OF SCIENCE AND TECHNOLOGY
SUCCEED WE MUST
P.O. BOX 1410, MBARARA

i) Likes/dislikes about the intervention,
ii) Anticipate benefits
iii) TB patients that can benefit most from mobile money-delivered transport assistance to the clinic?
iv) TB patients that benefit most from mobile money-delivered incentive to motivate medication adherence?
v) Amount of transport/incentive that would be sufficient
vi) Anticipated challenges in using mobile money to provide transport to TB clinic, and motivate medication adherence (**Probes**: *shared phones, possibility of TB status disclosure, access to mobile money agents, competing interests e.g. using the money for other issues*)
vii) Solutions to the identified challenges

Is there anything else you would like to tell me about the use of mobile money financial incentives to support TB patients?

Perceptions of SMS reminders

Read: I would now like to ask you some questions about the SMS texts that we will be sending TB patients to remind them take medication.

How do you feel about an intervention that utilizes SMS texts to remind medication adherence?
i) Likes/dislikes about the intervention,
ii) Anticipated benefits,
iii) Anticipated challenges (probe: *un authorized access, shared phones, phone functionality*)
iv) Solutions to the identified challenges.

Is there anything else you would like to tell me about the use of SMS reminders to encourage TB medication adherence.

Perceptions of Electronic Monitoring

Read: *I would now like to ask you some questions about the Wisepill device that will be sending us signals when a TB patient opens it to take medications.*

How do you feel about an intervention that utilizes the Wisepill device for supporting TB patients to take their medication?
i. Likes/dislikes about the intervention,
ii. Anticipated benefits
iii. Anticipated challenges (*probe: worries of unintended disclosure, being seen using the device, being known you open or not opens the device to take medication.*
iv. Solutions to the identified challenges?

RESEARCH ETHICS COMMITTEE MBARARA UNIVERSITY OF SCIENCE AND TECHNOLOGY P.O. BOX ... MBARARA

Is there anything else you would like to tell me about the use of the Wisepill device for monitoring how TB patients take their medication?

Perceptions of hair cutting

Read: *As part of this study, we will need to analyse patients' head hair to understand how the patients' has been taking medication. This will require patients not to cut their hair for at least a month before the first two months and at least a month before the end of the next four months of the study. Research assistants will cut a small thatch of hair (10-25 strands/0.02 ng/mg) (from the research office or the participants' place of choice) close to the occipital scalp. I would like to ask you about this approach.*

How do you feel about an intervention that requires you not to grow your hair for at least a month and later cuts a small sample of this hair (10-25 strands) to understand you medication taking?

What do you dislike and/or like in this approach?

What challenges do you anticipate in this approach? Probe: negative perceptions, where to cut it from, who to cut it, need to grow hair for at least a month?

How can the identified challenges be addressed?

Do you have any questions for me? *We have finished this discussion. Thank you very much for offering to talk to us.*

RESEARCH ETHICS COMMITTEE
MBARARA UNIVERSITY OF SCIENCE AND TECHNOLOGY
SUCCEED WE MUST
P.O. BOX 1410, MBARARA
