# Supplementary material for: Digital Adherence Technologies and Mobile Money Incentives for Management of Tuberculosis Medication Among People Living With Tuberculosis: Mixed Methods Formative Study
Source: JMIR Form Res. 2023 Apr 12;7:e45301. doi: 10.2196/45301 (PMC10134020; doi:10.2196/45301)
Supplement: Multimedia Appendix 2 [file formative_v7i1e45301_app2.pdf]

Participant ID__ __ __	Date __ __/__ __/__ __ __ __
Staff initials __ __ __	☐ Enrollment ☐ Interim

Demographics and health

Instructions: To be completed at enrollment, referencing the participant's medical chart in the TB Clinic as needed. This form may also be used to document changes during the study (complete with only new information).

Demographics

1. Date of birth: __ __/__ __/__ __ __ __
   d d m m y y y y

2. Gender: ☐Male ☐ Female

3. Primary residence			
County	Sub-County	Parish	Village/LC1
4. Secondary residence (*check here if not applicable*☐)			
County	Sub-County	Parish	Village/LC1
5. Would you call your residence a town or a rural area?			
Town ☐	Rural ☐		

6. Highest level of education achieved:

   ☐None ☐P1-P7 ☐ O' Level ☐ A' Level ☐ Tertiary

5. Literacy

RESEARCH ETHICS COMMITTEE MBARARA UNIVERSITY OF SCIENCE AND TECHNOLOGY SUCCEED WE MUST P.O. BOX 1410, MBARARA

	English	Runyankole
Able to speak	☐Yes ☐No	☐Yes ☐No
Able to read (*refer to language card*)	☐Yes ☐No	☐Yes ☐No

Cell phone use

Instructions: To be completed at enrollment and any time this information changes (complete with only new information).

		SIM registration verified
1. Preferred cell phone number	__ __ __ __ __ __ __ __ __ __	☐
2. Alternate cell phone numbers (check here if none ☐)	__ __ __ __ __ __ __ __ __ __<br>__ __ __ __ __ __ __ __ __ __	☐<br>☐

3. Who else uses your telephone?

   ☐Spouse ☐ Other family member ☐ Friend ☐ Neighbor

   ☐ Other (please specify)--

RESEARCH ETHICS [illegible]
MBARARA UNIVERSITY OF SCIENCE AND TECHNOLOGY
SUCCEED WE [illegible]
P.O. BOX [illegible]

Instructions: Complete the remainder of this form for participants in the intervention arms only.

Participant ID MBW0__ __ __	Date __ __/__ __/__ __ __ __
Staff initials __ __ __	Study arm: Arm A<br>Arm B<br>Control<br>Social Supporter
Enrollment Interim	

1. How often do you check your phone for SMS messages in a typical week?

☐ Never
☐ Less often
☐ More often

2. Have you ever been delayed from your usual schedule of checking your SMS messages because of any of the following in the last week?

☐ Your phone wasn't charged?
☐ Your SIM card wasn't in your phone?
☐ You did not have adequate cell signal?
☐ Your phone was not functioning?
☐ Someone else (i.e. family member who you share the phone with) had your phone at the time?
☐ Any other reason why you were unable to check your phone for SMS messages?

3. Language preference for SMS: ☐Runyankole ☐English

4. Preferred frequency of receiving SMS ☐ Daily ☐ Weekly

5. Preferred content for the SMS reminders (max 140 characters).*Participants not wishing to personalize content will receive the message, "This is your reminder". Add additional pages as needed.*

----

6. Which barriers do you anticipate to experience in receiving SMS reminders for TB medication?

☐ Charging the phone

☐ Message being seen by people you are not comfortable with

☐ Other (Specify)

....

....

RESEARCH ETHICS COMMITTEE
MBARARA UNIVERSITY OF SCIENCE AND TECHNOLOGY
SUCCEED WE MUST
P.O. BOX 1410, MBARARA

7. Which barriers do you anticipate in receiving mobile money for transport to the clinic and medication motivation? ....
....

Health

Tuberculosis

1. TB medication regimen (from clinical records)

☐ Isoniazid ☐ rifampicin ☐ pyrazinamide ☐ ethambutol ☐ Other

2. Type of TB infection

☐ Pulmonary, ☐ other

3. Date of initiating TB treating____________________

4. Duration of TB treatment planned_____________________

HIV/AIDS HIV Status ☐Negative ☐Positive

1. How many times do you take your ART medication in a day?

   ☐ Once a day

   ☐ Twice a day

   ☐ Sometimes once, or twice

2. *<for a patient>* I find it easier to take TB drugs than ARTs
☐ Strongly agree ☐ Agree ☐ Disagree ☐ Strongly disagree

TB Medication Taking Behavior

1. Which of the following is true about your TB medication taking behavior?
☐ Often take pills on the exact time prescribed by the Doctor? ☐ Sometimes delays taking medication ☐ Sometimes misses taking pills
Pease give reasons for your answer...

2. Which of the following explains why you sometimes miss or delay taking your medication? Tick all that apply.
☐ Forgets that it is time to take medication ☐ lacks transport to pick medication from the clinic ☐ I don't think taking medication on time is important ☐ others (please specify)

3. Do you think that being in a study that sends you SMS reminders to take your TB medication would help you take your medication in time?
   ☐ Yes ☐ No
   If yes, why…………………………………………..
4. Do you think that giving you a device that monitors and reports to us how you take your medication would help you take your medication in time?
   ☐ Yes ☐ No
   If yes, why…………………………………………..
5. Do you think that being in a study that sends you monthly mobile money incentives as transport to the clinic would motivate you take your medication?
   ☐ Yes ☐ No
   If yes, why…………………………………………..

RESEARCH ETHICS COM[illegible]
MBARARA UNIVERSITY OF SCIENCE AND TECHNOLOGY
SUCCEED WE MUST
P.O. BOX 1410, MB[illegible]
